# Newborn screening for sickle cell disease in Caluquembe, southwestern Angola, 2024–2025

**DOI:** 10.1371/journal.pone.0335720

**Published:** 2025-10-30

**Authors:** Jasmine J. Su, Vasco S. Kupua, Daniel Cummings, Kathryn H. Jacobsen

**Affiliations:** 1 Hospital Evangélico de Caluquembe, Caluquembe, Angola; 2 Department of Health Studies, University of Richmond, Richmond, Virginia, United States of America; Versiti Blood Research Institute, United States of America

## Abstract

**Objectives:**

Angola is one of the countries with the highest prevalence of sickle cell disease (SCD). Neonatal SCD testing is recommended by the Angolan government, but it is not routinely performed. Nearly all previous studies of SCD have been conducted in cities. We implemented a neonatal SCD screening program in a referral hospital in Huíla province to examine the epidemiology of SCD in this Umbundu-speaking population and to demonstrate the feasibility of using point-of-care (POC) tests for SCD in a rural setting.

**Methods:**

Between October 2024 and February 2025, we screened 353 infants less than one month old at Hospital Evangélico de Caluquembe for the hemoglobin S (HbS) gene using HemoTypeSC rapid diagnostic tests. We also reviewed all pediatric outpatient visits from 2024 to identify newly-diagnosed SCD cases.

**Results:**

Twenty-one (6.0%) of the 353 neonates had sickle cell trait (HbAS); none had SCD (HbSS). The outpatient register review identified 26 incident cases of SCD.

**Conclusions:**

The prevalence of HbS is lower in Caluquembe than in Luanda and Cabinda, but the combined results of our newborn screening and pediatric records provide evidence that there is a burden of disease from SCD in Caluquembe and the surrounding areas. Sickle cell screening and treatment programs should be available in all high-burden areas, not just large cities. The per-test costs may still be too expensive for universal newborn screening to be scaled up nationwide, but our pilot study demonstrates that POC tests can be a cost-effective method that yields immediate results.

## Introduction

Sickle cell disease (SCD) is a genetic blood disorder that is caused by inheriting an abnormal hemoglobin subunit beta (HBB) gene from both parents. Sub-Saharan African countries have the world’s highest prevalence rates of SCD [1–3], and an estimated 50% of African children with SCD do not survive to their fifth birthdays [4–7]. SCD is understudied in Angola, a country in southwestern Africa with nearly 40 million residents [8]. The Cadernos de Saúde Materno-Infantil (maternal and child health notebooks) provided by the Angolan government to guide clinical care recommend that newborns receive eye ointment; vaccines against BCG, polio, and hepatitis B; and a heel prick test for SCD. However, many cases of SCD remain undiagnosed in Angola due to limited access to diagnostic tools, and many individuals who are diagnosed at any age are unable to access specialized care [9].

The HBB gene is responsible for the production of β-globin, which is one component of the hemoglobin protein that carries oxygen within red blood cells. The most frequently occurring HBB gene variant is hemoglobin S (HbS), which can cause red blood cells to become rigid and misshapen [10]. Individuals with one normal hemoglobin gene (hemoglobin A, abbreviated as HbA) and one HbS gene are carriers of the sickle cell trait (HbAS), and they can pass the HBB gene to their offspring. Individuals with two HbS genes have homozygous (HbSS) SCD and may experience anemia, jaundice, fatigue, swelling of hands and feet, frequent infections, and occasional vaso-occlusive crises that cause severe pain [11]. These episodes can damage the liver, kidneys, heart, lungs, eyes, and other organs, and they can cause strokes, organ failure, sepsis, and death.

Neonatal screening for SCD with rapid tests can improve health outcomes for people with SCD by enabling them to access the health services they need to manage their condition before complications occur [12–14]. However, newborn screening programs in low-income countries have been reported to be prohibitively expensive and technologically complicated, especially in rural areas [15]. There is a need for additional studies about the feasibility of scaling up SCD screening of newborns across Africa and the cost-effectiveness of such efforts [16–20].

Angola is an ethnically diverse country where more than 40 languages are spoken in addition to the official language, Portuguese. Most tribal groups have remained in specific geographical locations for many generations, so the prevalence of SCD in different populations within Angola may vary significantly. Angola is estimated to have a higher incidence and prevalence of SCD than its regional neighbors [1,2] , which is why the Cadernos de Saúde Materno-Infantil recommends sickle cell screening for all newborns. However, previous studies of SCD testing, treatment, and patient experiences in Angola have recruited participants from only a few places: the capital region of Luanda [9,21–37]; the exclave of Cabinda, which is a small province that is physically separated from the rest of Angola by a strip of land belonging to the Democratic Republic of the Congo [38–40]; and Huambo, in central Angola [41]. Results from those locations are not necessarily generalizable to rural areas or other sociodemographic groups.

We sought to expand knowledge of SCD burden in diverse Angolan populations by conducting a study in Caluquembe, a rural municipality in Huíla province in southwestern Angola, where Umbundu is the primary language [42]. Our study had two main aims. The first objective was to examine the incidence and prevalence of SCD in newborns at Hospital Evangélico de Caluquembe. This information will contribute to the hospital’s efforts to identify unmet needs for health services in the populations it serves and increase access to preventive, diagnostic, and therapeutic care. The second objective was to test the feasibility of using point of care (POC) testing to screen for SCD in our hospital.

The gold standard for SCD diagnosis is gel electrophoresis testing  [43], but this is largely unavailable to patients in Caluquembe. At present, the nearest facility where residents of Caluquembe can access gel electrophoresis tests is a private clinic in Lubango, the regional capital, which is 200 kilometers away [44]. A variety of new POC diagnostic tests have made it feasible to conduct SCD testing in places where resource-intensive methods like gel electrophoresis, high-performance liquid chromatography, and DNA analysis are not available [43]. Demonstrating the feasibility of low-cost POC testing for SCD in our part of rural Angola has relevance for other places in sub-Saharan Africa and other world regions where recommended neonatal screening tests are not yet being routinely provided to all eligible patients.

## Materials and methods

Caluquembe is a rural municipality with around 200,000 residents that is located 200 km northeast of the provincial capital of Lubango and 210 km southwest of the city of Huambo. Hospital Evangélico de Caluquembe, a 200-bed facility that serves a catchment area of about 500,000 people, is the only facility between Lubango and Huambo that offers emergency surgical services. About half of babies in Angola are born at hospitals or birth centers, but in Huíla province up to 70% of births occur at home [45]. Many of the women who choose to deliver at a healthcare facility do so because they are experiencing complications during labor or have a high risk of complications during labor and delivery. In 2024, 508 of the 1258 babies born at Hospital Evangélico de Caluquembe required surgical interventions such as forceps delivery, vacuum extraction, or cesarean section.

Several private clinics and public hospitals and clinics in the area offer antenatal consultations and basic maternal, obstetric, and neonatal care services. Private providers charge fees for all services. Public providers deliver antenatal services and perform uncomplicated deliveries for free, but family members may be required to purchase medicines and other supplies if needed materials are not available at the facilities. All these facilities refer complicated cases to Hospital Evangélico de Caluquembe, a publicly subsidized private hospital that provides comprehensive emergency and obstetric care, including laboratory testing, imaging, and surgical delivery options that are not available at other regional healthcare facilities. As a referral center, Caluquembe charges most patients for services, but it provides care to all patients seeking emergency care even if they are not able to pay.

The Ethics Review Committee of Hospital Evangélico de Caluquembe reviewed and approved the research protocol for this study in July 2024. All infants less than one month old who were delivered vaginally or via Cesarean section at Caluquembe, neonates who were born elsewhere and then admitted to the hospital, neonates whose mothers were admitted to the maternity ward (even if the newborn was not), and neonates whose siblings (typically twins) were admitted to Caluquembe were eligible for inclusion. Eligible neonates were identified daily between October 2024 and February 2025 following delivery or admission, and their parents were invited by the lab team to participate in the study. The consent process emphasized that participation was voluntary and that all patients and their family members would receive the same quality of care regardless of their decision about whether to participate in this study. (The siblings of SCD patients at Caluquembe are offered free testing for HbSS as part of family care for children with SCD; none of the newborns enrolled in the study were siblings of SCD patients being treated at our facility.) Consent was obtained verbally from the mother of eligible newborns and was witnessed by a staff person who was not a member of the research team. The study database did not include identifiable patient information such as names and addresses. To further ensure that HbS test results could not be linked to any individuals, birthdates and birthweights were removed from the public dataset.

After receiving consent from the babies’ parents, blood was drawn via heel prick. The HemoTypeSC rapid test for sickle cell trait and disease was used to identify the genotypes of study participants [46]. Test results are available in 10 minutes, so the entire process from specimen collection through results takes less than 15 minutes. The rapid test has a sensitivity and specificity of nearly 100% when compared to more time-intensive laboratory tests [9,47,48]. Three lab technicians at the hospital were trained on the use of the test kits. The process of collecting the blood sample, doing the HemoType SC rapid test, and interpreting results was carefully supervised by study personnel until each technician had completed at least 10 tests. Random checks of testing protocol and data collection were conducted by the assistant lab director. All tests with indeterminate results were repeated and then reviewed by the assistant lab director and a study physician.

In addition to prospectively testing newborns for HbS, we conducted a review of the pediatric outpatient register to identify patients who had been diagnosed with SCD for the first time between January and December 2024 at Hospital Evangélico de Caluquembe. We used outpatient records rather than inpatient files because our quality control checks found that SCD status was often not noted on the charts of known SCD patients who had been admitted to the hospital for malaria, osteomyelitis, or other complications of SCD. We recognize that it is likely that our review of outpatient test results significantly underestimates the prevalence of SCD, since testing was conducted only on outpatients with symptoms consistent with SCD. However, our goal for this part of the study was not to measure the prevalence of SCD, merely to confirm that SCD is present in the communities the hospital serves.

We used simple proportions and counts to examine our results. We used the mid-p exact 95% confidence interval (CI) for proportions. The Fisher exact test with a significance level of α = 0.05 was used to compare the prevalence by sex.

## Results

Of the 353 newborns who were tested for HbS, 332 (94%) had HbAA genotype and were not sickle cell carriers, 21 (6% with a 95% CI of 4–9%) had HbAS genotype and were sickle cell carriers, and 0 (0% with a 95% CI of 0–1%) had the HbSS genotype that causes SCD. In total 13 of 173 (8%) male participants and 8 of 180 (4%) female participants had HbAS. The difference in prevalence by sex was not statistically significant (p = 0.31). There was no difference in birthweight by HbS status overall (p = 0.30), for boys (p = 0.45), or for girls (p = 0.63).

The review of the outpatient register from 5970 total pediatric consultations identified 26 children who were diagnosed with SCD at the hospital during the 2024 calendar year. The 26 incident cases included 16 male and 10 female patients between the ages of 8 months and 14 years old. Of these patients, 19 were from Caluquembe municipality and 7 were from other municipalities.

The combined evidence from the newborns who were identified as HbS carriers and the children with incident HbSS diagnoses provides evidence that SCD is present in the catchment area. These results also demonstrate that POC rapid diagnostic test (RDT) kits can be used successfully in rural Angola to provide nearly immediate identification of sickle cell carriers.

## Discussion

Our neonatal screening program found a 6.0% prevalence of HbS among newborns who did not have siblings with confirmed SCD. The prevalence of HbS in our study population is lower than the rate reported in newborn screening studies in Luanda and Cabinda. At Bengo General Hospital in 2014–2016, 82 (22.8%) of 359 newborns had HbAS and 12 (3.3%) had HbSS [22]. At ten maternity hospitals and vaccination clinics in Luanda, 442 (21.9%) of 1958 infants had HbAS and 34 (1.7%) had HbSS [9,35]. At sixteen birth centers that participated in the Angola Sickle Cell Initiative in Luanda (starting in 2011) and Cabinda (starting in 2012), 1488 (1.7%) of more than 85,000 newborns had HbSS [30]. At two maternity hospitals in Luanda in 2011–2013, Maternidade Lucrecia Paim and Maternidade Augusto Ngangula, 7666 (21.0%) of 36,453 newborns had HbAS and 550 (1.5%) had HbSS [28]. At the obstetric center of the Primero de Maio hospital in Cabinda, 31 (19.5%) of 159 newborns had HbAS and 2 (1.3%) had HbSS [39]. At Hospital Materno Infantil Dr Manuel Pedro Azancot de Menezes in Luanda in 2023–2024, 1719 (18.9%) of 9074 newborns had HbAS and 121 (1.3%) had HbSS [23].

Given the ethnic diversity in Angola, results of population-based studies of hemoglobinopathies and other genetic disorders in one location may not be generalizable nationwide. Caluquembe is an Umbundu-speaking region with limited in-migration, so the lower rate of SCD in this area may be evidence of a lower rate in this ethnic population than in those from Luanda and other regions. Differences in the prevalence of SCD among various ethnic and tribal groups have been previously reported in countries such as Guinea-Bissau [49] and Sudan [50]. More research is required to confirm the variability of the prevalence of HbS in diverse geographic and ethnic populations across Angola.

While none of the newborns who participated in our neonatal screening program had HbSS, the hospital’s outpatient register showed that 26 children were diagnosed with SCD in 2024. If newborn screening had been available to these children when they were newborns, they could have been diagnosed at much younger ages and received medical care to manage the condition and prevent complications. All diagnoses after the neonatal period represent missed opportunities for earlier diagnosis.

The combined results of our newborn screening and pediatric records provide evidence that this region of southwestern Angola bears a burden from SCD, both in Caluquembe municipality and the surrounding areas, even if the prevalence of HbS in this region is lower than in some other areas of Angola. While hydroxyurea is not available in Caluquembe municipality, all other standard treatments are able to be provided locally. It is important for newborns in Caluquembe to be tested for HbSS and for all SCD patients to have access to clinical care, ideally including hydroxyurea.

Patients with SCD who are treated at Hospital Evangélico de Caluquembe typically have bimonthly checkups that include a free clinical consultation and a hemoglobin test that costs about $1.25. However, that is not the full cost of the checkup. The average cost of transportation for the patient and at least one caregiver is at least $6 per clinic visit. If hydroxyurea is prescribed, a caregiver must travel from Caluquembe to Lubango, with transportation costs averaging $7 per trip, and then pay at least $30 per month for the medication. Given that the average household income in the areas around Caluquembe is about $10 to $30 total per month, SCD is a substantial financial burden on affected families. Since many households affected by SCD are home to more than one member with the genetic disorder, these costs are even more burdensome. Patients may have no choice but to forego medical care when the household cannot afford the direct and indirect costs of health care.

The Angolan government does not provide free or low-cost HbS test kits to hospitals, and the few private vendors in Angola that import HbS test kits are not always able to keep them in stock. At the time of our study, test kits cost about $1–2 each when purchased from the manufacturer, and about 8–10 times more when purchased from in-country vendors. Our screening program was able to be implemented because we purchased HbS tests at a discounted rate outside of Angola and then brought them into the country legally. We were able to demonstrate the logistic feasibility of hospitals far from the capital city procuring rapid test kits and using them to provide quick results to parents of newborns. POC testing is easy to use — especially compared to gel electrophoresis, which is more expensive and complicated than RDTs and takes significantly more time to provide test results — and the immediate results POC testing yields allow for early initiation of clinical interventions.

Even though the per-test costs of POC for HbS are still too high to allow for universal newborn screening for SCD across Angola at this time [9,18,28,39], we have demonstrated ways to reduce the costs of testing programs. We kept the personnel costs associated with HbS screening relatively low by training several already-skilled staff members to perform the neonatal sickle cell screening tests. Our healthcare team members are also skilled at providing counseling to the families of babies who test positive for HbS and delivering medical care to children with HbSS. Even though we did not identify any sickle cell disease (HbSS) cases among the newborns in our pilot screening program, we are prepared to provide early intervention for future newborns who are diagnosed at the hospital.

There were several limitations to our study. Our participation rate among eligible neonates was very high, but with only 353 participants we had limited power to precisely quantify the prevalence of HbSS in this region and compare the prevalence rate to rates reported from elsewhere in Angola. Our study only sampled neonates born at or brought to our hospital. Babies delivered at hospitals may have systematically different socioeconomic and other characteristics than those who are born at home. We hope, in the future, to be able to test the cost-effectiveness of screening babies who were born at home for HbS in locations outside of hospitals. This type of screening program would be conducted in collaboration with clinics in partner communities, perhaps as part of a combined effort to deliver hepatitis B vaccines to rural newborns. There is also a need for additional studies that compare test costs and feasibility in rural versus urban areas.

Despite these limitations, our study contributes to expanding knowledge about the sickle cell burden in Angola and providing evidence that it is possible to conduct newborn screening tests even in remote areas of low-income countries. Expanded SCD testing will enable improved accuracy of maps of the local burden of SCD in Angola and elsewhere, and that will facilitate evidence-based prioritization and allocation of resources for SCD prevention, screening, and management. POC tests are low-cost and convenient. We encourage health centers across Angola and beyond—in any places where SCD is a known burden—to begin or expand neonatal sickle cell screening programs, as resources allow, and publish their findings so that they can be incorporated into meta-analyses like the Global Burden of Disease Study that will improve the accuracy of estimates of SCD prevalence across the sub-Saharan African region and globally [2].

## Supporting information

S1 File Data file. (XLSX)

S2 File Abstract in Portuguese. (DOCX)

S3 File Inclusivity in global research questionnaire. (DOCX)
