# Supplementary material for: Newborn screening for sickle cell disease in Caluquembe, southwestern Angola, 2024–2025
Source: PLoS One. 2025 Oct 30;20(10):e0335720. doi: 10.1371/journal.pone.0335720 (PMC12574841; doi:10.1371/journal.pone.0335720)
Supplement: S2 File — (DOCX) [file pone.0335720.s002.docx]

**Triagem neonatal da anemia falciforme**

**em Caluquembe, sudoeste de Angola, 2024–2025**

**Resumo**

Objetivos: Angola é um dos países com maior prevalência de anemia falciforme (AF). O teste de AF neonatal é recomendado pelo governo angolano, mas não é realizado rotineiramente. Quase todos os estudos anteriores sobre AF foram realizados na capital. Implementamos um programa de triagem de AF neonatal em um hospital de referência na província da Huíla para examinar a epidemiologia da AF nessa população de língua umbundu e demonstrar a viabilidade do uso de testes de ponto de atendimento.

Métodos: Entre outubro de 2024 e fevereiro de 2025, testamos 353 recém-nascidos (bebês com menos de um mês de idade) no Hospital Evangélico de Caluquembe para o gene da hemoglobina S (HbS) usando testes de diagnóstico rápido HemoTypeSC. Também realizamos uma revisão de todos os registros ambulatoriais pediátricos de 2024 para identificar AF recém-diagnosticados.

Resultados: Vinte e um (6,0%) dos 353 neonatos apresentavam traço falciforme (HbAS). Nenhum apresentava anemia falciforme (HbSS). A revisão dos prontuários médicos identificou 26 casos incidentes de AF.

Conclusões: A prevalência de HbS é menor em Caluquembe do que em Luanda e Cabinda, mas os resultados combinados do nosso rastreio neonatal e dos registos pediátricos evidenciam a existência de uma carga de doença por AF em Caluquembe e áreas circundantes. As normas governamentais exigem que os programas de rastreio e tratamento da anemia falciforme estejam disponíveis em todas as áreas com elevada incidência, não apenas nas grandes cidades, embora os custos por teste possam ainda ser demasiado elevados para serem expandidos a nível nacional. O nosso estudo piloto demonstra que os testes de ponto de atendimento podem ser um método rentável e com resultados imediatos.

**Palavras-chave:** hemoglobina S; traço falciforme; doença falciforme; triagem neonatal; serviços de diagnóstico; Angola
